# Emergence of a Novel, Phenotypically Difficult‐to‐Detect Vancomycin‐Resistant *Enterococcus faecium* Clone (ST117/CT7799)

**DOI:** 10.1002/mbo3.70393

**Published:** 2026-08-31

**Authors:** Patrick Forstner, Christine Uitz, Johanna Dabernig‐Heinz, Gabriel E. Wagner, Jennifer Bender, Martin Fischer, David Siebenhofer, Guido Werner, Tobias Busche, Levin Joe Klages, Christian Rückert‐Reed, Ivo Steinmetz, Karl Dichtl

**Affiliations:** ^1^ Diagnostic and Research Institute of Hygiene, Microbiology and Environmental Medicine Medical University of Graz Graz Austria; ^2^ Division of Nosocomial Pathogens and Antibiotic Resistances, Department of Infectious Diseases Robert Koch Institute Wernigerode Germany; ^3^ German National Reference Centre for Staphylococci and Enterococci Wernigerode Germany; ^4^ Medical Faculty OWL Bielefeld University Bielefeld Germany; ^5^ Center for Biotechnology Bielefeld University Bielefeld Germany; ^6^ Institute of Laboratory Medicine and Microbiology, Protestant Hospital of Bethel Foundation, Campus Bielefeld‐Bethel University Hospital OWL of Bielefeld University Bielefeld Germany

**Keywords:** antimicrobial susceptibility testing, *Enterococcus faecium*, ST117/CT7799, vanB, vancomycin resistance, VRE

## Abstract

A significant increase of vancomycin‐resistant *Enterococcus faecium* (VREfm) infections was observed in South‐Eastern Austria since 2024. The prolonged outbreak is caused by a novel *vanB‐*VREfm clone (ST117/CT7799, “VREfm_styr_”). This study characterizes the atypical difficult‐to‐detect resistance phenotype and assesses the genomic relatedness of the isolates. Patient and outbreak characteristics were investigated including whole genome sequencing of the isolates. Sensitivity of broth microdilution (BMD), gradient tests (GT), disk diffusion (DD), and automated susceptibility testing (VITEK2) was compared. The performance of commercial screening media was evaluated. From sporadic detections in early 2024 case numbers began to rise during the year. In 30/31 (97%) of all cases, intra‐hospital transmission was considered likely and an association with invasive procedures was identified in most cases. Core genome multilocus sequence typing revealed only six allelic differences between VREfm_styr_ isolates collected in a 12‐month period, all belonging to the *E. faecium* ST117/CT7799 lineage. BMD detected vancomycin resistance (MIC > 4 mg/L) in no more than 16/31 (52%) of isolates after 24 h incubation, while GT and DD misclassified all isolates. Only prolonged incubation improved the performance of these assays. VITEK2 analysis, however, correctly classified all 31 isolates. Of four commercially available VRE‐screening agars, only one was capable of detecting VREfm_styr_ after 24 h incubation. The emergence and clonal dissemination of VREfm ST117/CT7799 reveals a serious diagnostic gap as commonly used diagnostic algorithms fail to reliably detect this resistance phenotype. Our findings should help to further evaluate the true geographical distribution and clinical significance of this novel VREfm clone.

## Graphical abstract

A newly emerged vanB‐VREfm clone (ST117/CT7799) causes an ongoing outbreak in southeastern Austria. The cluster is highly homogenous, even for isolates sampled months apart. Resistance is easily missed via disk diffusion, gradient tests, and microdilution. VITEK 2 testing reliably detects vancomycin‐resistance. Most commercial screening agars fail to identify this clone.

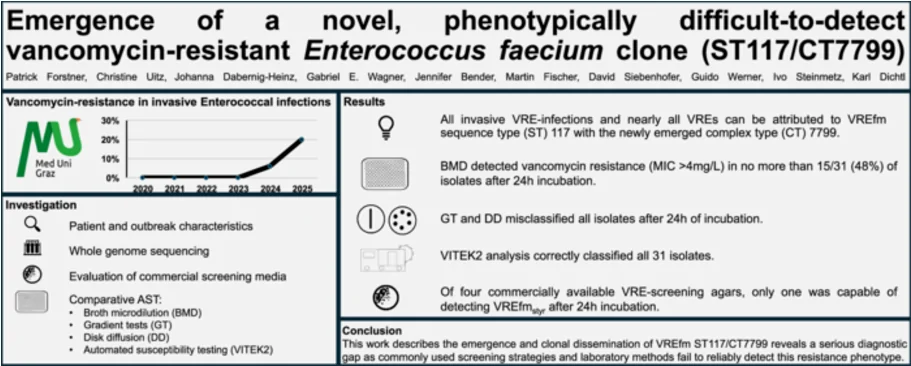

## 1 Introduction

Enterococci are gram‐positive bacteria that colonize the intestinal tract of animals, including that of humans (Bender et al. 2022; Cetinkaya et al. 2000). Although mostly being considered harmless commensals, they can cause localized as well as extensive and systemic infections (Bender et al. 2022; Cetinkaya et al. 2000). Within the genus *Enterococcus*, *Enterococcus faecalis*, and *Enterococcus faecium* (Efm) are considered the most relevant disease‐causing species (Bender et al. 2022; Cetinkaya et al. 2000). Particularly the latter is known for acquisition of resistance against penicillins and, with increasing rates, glycopeptides (Bender et al. 2022). This bactericidal antibiotic class binds to the peptidoglycan precursor and—by doing so—inhibits its incorporation into the cell wall (Blaskovich et al. 2018). Although vancomycin was first introduced in 1955, the first description of vancomycin‐resistant enterococci did not occur until over 20 years later (McCormick et al. 1955; Leclercq et al. 1988; Uttley et al. 1988). Enterococci harboring the *vanB* operon, which has been the most prevalent genotype among vancomycin‐resistant Efm (VREfm) in several European countries over the last years, display a more variable phenotypic resistance compared to *vanA*‐carrying enterococci (Grabsch et al. 2008; Klare et al. 2019; Werner et al. 2012; Knudsen et al. 2025; Brajerova et al. 2025).

The increasing prevalence of VREfm is a major challenge for public health worldwide (World Health Organization 2024). Known for its capacity to cause opportunistic infections and nosocomial outbreaks, it has been classified as a high‐priority pathogen in the WHO 2024 Bacterial Priority Pathogens List (World Health Organization 2024). Even if treated adequately, invasive infections such as sepsis are associated with prolonged hospitalization and increased mortality (Prematunge et al. 2016).

According to ECDC, the mean vancomycin resistance rate in European Efm isolates is 20% (European Centre for Disease Prevention and Control 2024). In 2023, the Austrian national action program on antibiotic resistance reported a vancomycin resistance rate of 3% among invasive Efm isolates (Bundesministerium für Soziales GP und K BMSGPK 2024). After years without any invasive VREfm infections, our laboratory, which provides microbiological diagnostics for the University Hospital of Graz (Austria) and more than 1600 medical practices in South‐Eastern Austria, observed a sharp increase in 2024: the VREfm‐rate in Efm‐positive blood cultures increased to 6% in 2024 and to 20% in the first half of 2025. By June 2025, 31 VREfm were isolated and included in this study. All invasive VRE‐infections and nearly all VREs can be attributed to the newly emerged complex type (CT) 7799 and VREfm sequence type (ST) 117 (Abdelbary et al. 2019; Caplunik‐Pratsch et al. 2024).

To our knowledge this is the first description of this novel VREfm strain. We present epidemiological insight into an ongoing outbreak, discuss the strain's microbiological characteristics, and highlight the diagnostic challenges caused by its difficult‐to‐detect resistance phenotype.

## 2 Methods

An overview of media, antimicrobial susceptibility testing (AST) methods (automated and manual), reagents, laboratory instrumentation, and control strains is provided in Supporting Information S2: Table S1. The study included the first cases (*n* = 31) in which a VREfm strain displaying a novel AST phenotype was dertected. The corresponding 31 retrospective, non‐duplicate VREfm isolates were recovered from routine clinical specimens submitted for infection diagnostics, not screening, between February 2024 and June 2025. They comprised blood cultures (*n* = 5), wound swabs (*n* = 6), samples from invasive procedures (e.g., fluid aspirate; *n* = 5), and urine samples (*n* = 15). All isolates identified in routine diagnostics as VREfm and demonstrating the characteristic phenotype under investigation were consecutively included. Whole genome sequencing (WGS) demonstrated that isolates sharing the characteristic VRE_styr_ AST phenotype formed a highly concordant clonal lineage, allowing subsequent isolates with the same distinctive susceptibility profile to be assigned without further sequencing. In addition to isolates of the first 19 cases, one isolate of the very first case, which was subjected to in vitro long‐time vancomycin exposure, was sequenced resulting in sequencing data of a total of 20 isolates belonging to the local cluster.

Specimen and patient characteristics are summarized in Supporting Information S2: Table S2. Local controls were chosen from *Enterococcus faecium* blood stream infections. These were isolated at the same site at representative time points during the study period.

All isolates were stored at −80°C. Before batchwise (re‐)testing, the isolates were thawed and sub‐cultured overnight on blood agar at 35°C ± 2°C. All assays were independently read by two experienced microbiologists, and in case of disagreement, a third specialist was consulted. Susceptibility categorization was performed based on EUCAST clinical breakpoints (The European Committee on Antimicrobial Susceptibility Testing 2025). The BD Kiestra Total Lab‐Automation system was used for semiautomated inoculation of screening plates (10 µL of a 10^5^ CFU/mL suspension), incubation at 35°C ± 2°C, imaging, and zone diameter measurement.

DNA was extracted from pure cultures on blood agar after overnight incubation using the NucleoSpin Microbial DNA Kit (Macherey‐Nagel) and subsequent AXP wash for purification as described previously (Wagner et al. 2023). The DNA underwent WGS using both Illumina and Oxford Nanopore Technologies (WGS metrics specified in Supporting Information S2: Table S3). For short‐read (SR) sequencing, the library was prepared using the Nextera XT preparation kit and sequencing was performed on a Nextseq. 200 device with a P1 cartridge for a 150 basepairs (bp) paired‐end run. Raw SR data was de‐novo assembled in SeqSphere+ (Souvorov et al. 2018). After contamination checks in SeqSphere, the reads of one isolate (EF35) were mapped to a close reference genome (EF12) to remove a substantial portion of reads from another species (~40%) and assembly resumed with the filtered subset (Goig et al. 2020). Long‐read (LR) sequencing was carried out on an R.10.4.1 flow cell (ONT) from Oxford Nanopore technologies using the native barcoding kit (SQK‐NBD114.24). Basecalling was performed in Minknow using the up‐to‐date versions (at the respective time of sequencing) of dorado and basecalling models ‐m dna_r10.4.1_e8.2_400bps_sup@v5.0.0 and v5.2.0. Sequencing reads were assembled (flye v2.9.5) and polished (medaka v2.0.1) in analogy to Dabernig‐Heinz et al. (2024) (Supporting Information S2: Table S1), and the resulting genomes were genotyped using specific vancomycin‐targeted task templates, based on all relevant genes for vancomycin resistance detected by AMRfinder+ (Feldgarden et al. 2021), and the corresponding core genome multilocus sequence typing (cgMLST) scheme in SeqSphere+ (de Been et al. 2015). Genotyped reference strains were used as non‐cluster controls (Supporting Information S2: Table S1). Pilon was utilized to assess the per‐base deviations of the ONT assemblies from the respective SR reads per isolate. Blast+ and minimap2 were used to check completeness and identity of the transposon Tn*1549*, which is responsible for *vanB* vancomycin resistance (Grabsch et al. 2008). For analysis of rearrangements of Tn*1549* one representative genome of CT7799 (VREfm12) was annotated and analyzed with Geneious Prime 2023.1.1. The cross‐national surveillance platform MiGenomeSurv was used to delineate the novelty of CT7799 (miGenomeSurv‐Consortium 2021).

## 3 Results

### 3.1 Emergence of a Novel Difficult‐to‐Detect VRE Phenotype

**Figure 1:**
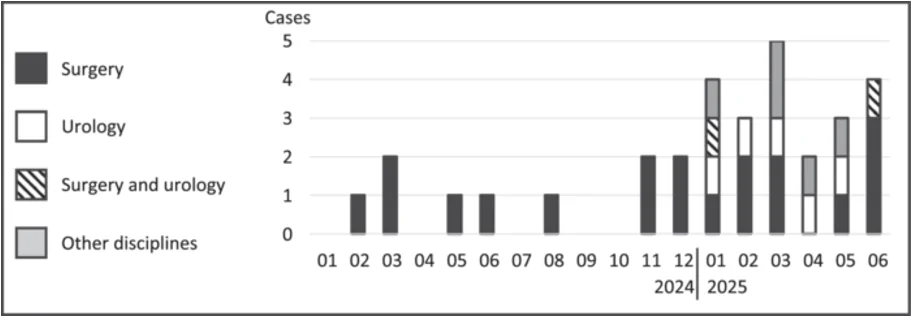
Outbreak timeline depicting newly diagnosed cases of VREfm_styr_ per month for the 31 isolates included in this study. Bars are color‐coded according to the clinical discipline where patients were treated.

In February 2024, VITEK2 susceptibility testing identified vancomycin resistance in a clinical *E. faecium* isolate, which remained undetected by disk diffusion (DD), gradient testing (GT), or broth microdilution (BMD) in routine diagnostics setting. Workflow reorganization to automated testing of all enterococci via VITEK2 and comprehensive re‐testing revealed five additional isolates with the same specific phenotype within the following months (Figure 1). In all six patients, samples were obtained not for screening purposes, but for infection diagnostics. *VanB*‐type resistance was confirmed by molecular analysis in all cases (Xpert *vanA*/*vanB*‐PCR, Cepheid AB, Solna, Sweden). A review of raw VITEK2 and Kiestra data from previous test results revealed no evidence of undetected enterococci with this specific phenotype in our laboratory before 2024. All cases were linked to invasive procedures in one hospital.

Initially, only sporadic cases were observed, however, from November 2024 onwards a continuous flow of new cases occurred (Figure 1). Between February 2024 and June 2025, 52 of 1079 *E. faecium* isolates subjected to AST in our laboratory were identified as VREfm (4.8%). The first 31 cases involving isolates with these distinctive phenotypic characteristics were subjected to a detailed analysis in order to investigate the outbreak. Most of these cases were still associated with surgical procedures that were performed at the same hospital. As a second group, urology patients, who had undergone double‐J catheterization, were identified. This procedure was performed in an operating room shared by urologists and surgeons. Investigations of the hospital infection control team, including environmental surface sampling of rooms and medical equipment failed to detect the VREfm strain. Virtually all isolates (30/31; 97%) were obtained from patients who had either been hospitalized for ≥ 4 days at the time of specimen collection (20/31; 65%) or had a history of inpatient care by the same healthcare provider within 6 months prior to VREfm detection (10/31; 32%).

**Figure 2:**
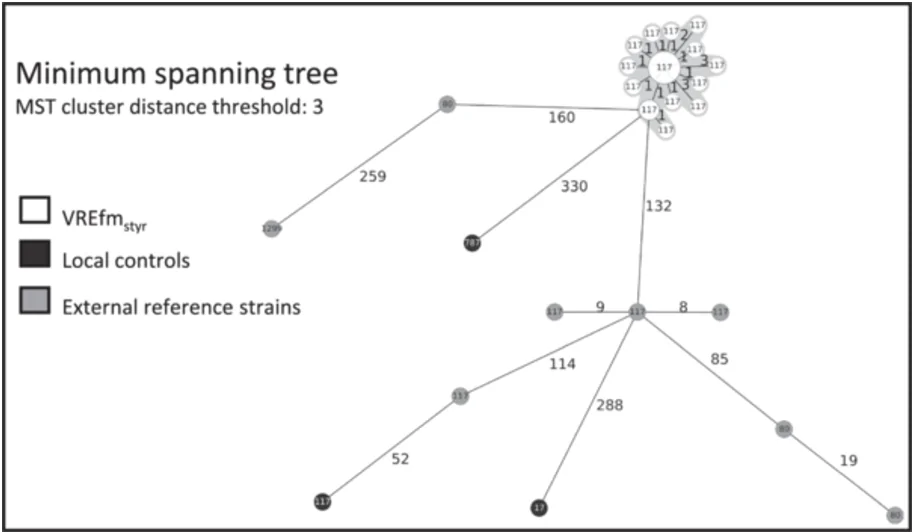
Minimum spanning tree of 30 *Enterococcus faecium* SR‐genomes based on 1423 targets using the *Enterococcus faecium* core genome multilocus sequence typing (cgMLST) scheme (de Been et al. 2015) created using default parameters in SeqSphere. All ST117/CT7799 outbreak isolates (VREfm_styr_, white) formed a single cluster with the chosen threshold of 3 allelic differences. Numbers on the lines represent allelic differences between the respective strains. Local contemporaneous controls are shown in black. RKI reference genomes from various STs, including ST117, are depicted in gray (see Supporting Information S2: Table S1).

WGS of LR and SR assemblies was concordant per isolate, meaning there were no differing cgMLST targets between assemblies of the same isolate. Additionally, only 11–16 Illumina‐supported base‐corrections per genome (~3 million basepairs) were found using Pilon, corresponding to a high consensus quality of the ONT‐only genomes (~Q50). The cgMLST analysis is exemplarily shown with Illumina based assemblies. WGS proved all 20 analyzed (non‐duplicate) isolates to belong to one outbreak (cluster threshold: 3) caused by a strain belonging to the *E. faecium* ST117/CT7799 lineage (Figure 2, Supporting Information S1: Figures S1 and S2). This clone will be referred to as VREfm_styr_ hereafter, named after the federal state of Styria, where it was first isolated. The cluster proved to be highly homogenous—even when comparing the first and the latest isolates sampled over 16 months apart—with a maximum of six allelic differences in cgMLST analysis based on 1423 loci. The closest local control strain, isolated at the same site and within the same timeframe, and the closest RKI reference strain differed by 159 and 142 alleles (Figure 2). According to the cross‐national surveillance platform MiGenomeSurv, the next closest relative still showed a distance of 76 alleles.

**Figure 3:**
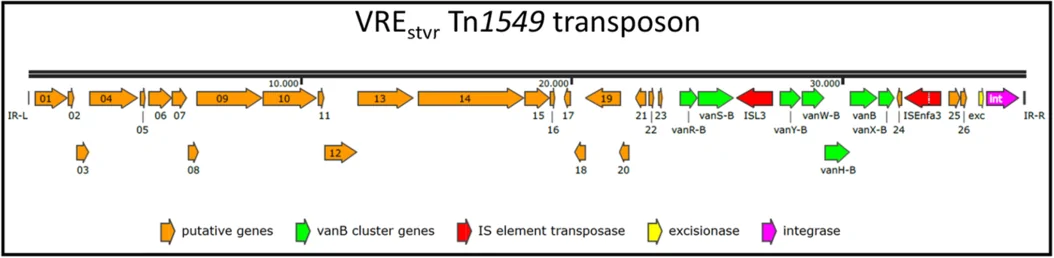
Structure of the Tn*1549* transposon of VRE_styr_. Scale bar indicates basepairs.

WGS revealed the presence of the complete sequence of the Tn*1549* transposon in both LR and SR genome assemblies. However, the sequence of the transposon was fragmented into three different genome contigs in the SR assemblies, which is why further genomic analysis of the chromosomally‐encoded transposon relied on the more continuous LR assemblies. Alignments of the Tn*1549* reference to LR assemblies demonstrated three matching blocks on the same contig, separated by two intervening regions of 1494 bp and 1417 bp in every LR‐based genome of CT7799 (Figure 3). Inspection of these intervening sequences in annotated genomes revealed an *ISL3*‐like element ISEfa11 family transposase in the first gap (directly after *vanS*‐B), and both an *IS3*‐like element IS*Enfa3* family transposase and helix‐turn‐helix domain‐containing protein in the second gap (directly after *vanX*‐B). This pattern was evident in one control isolate (EF27) and all outbreak isolates. In addition, we found an IS91 transposase inserted in the YodL domain‐containing protein upstream of the *vanB* cluster. Based on the classification by the Norwegian VRE study group (Al Rubaye et al. 2023), our *vanB* appears to be a mix between variant A (usually found in ST192) and variant B (usually found in ST17, albeit with the IS element inserted in the opposite direction). Given the insertion site in the *sir* gene, it stands to argue that our cluster is a representative of variant A that, by chance or selective pressure, has caught a second insertion element at nearly the same position as variant B. Interestingly, variant A was also found in the dominant outbreak strains in the Norwegian study.

Apart from the cgMLST targets typed for outbreak analysis (Figure 2), an additional analysis of LR genomes was conducted using a customized SeqSphere task‐template to screen all seven genes relevant to vancomycin resistance according to AMRFinderPlus (*vanB*, *vanY*‐B, vanW‐B, *vanH*‐B, *vanX*‐B, *vanR*‐B, and *vanS*‐B). This revealed identical sequences in all outbreak isolates and one control strain with a common vancomycin resistance phenotype (Table 1). The comparison to VRE strains from the German reference center revealed identical alleles for *vanB*, *vanH*‐B and *vanX*‐B, but differing alleles for *vanR‐*B, *vanY‐*B, and *vanW*‐B from the VREfm_styr_ genomes.

**Table 1:** Genotyping results and vancomycin resistance gene alleles of *E. faecium* isolates based on long‐read assemblies (AMRFinderPlus, SeqSphere + ).

Isolates	ST	CT	ST*	Van. res.	*vanB*	*vanH*‐B	*vanR*‐B	*vanS*‐B	*vanW*‐B	*vanX*‐B	*vanY*‐B
Outbreak strains											
EF12	117	7799	143	*vanB*	1	1	1	1	1	1	1
EF13	117	7799	143	*vanB*	1	1	1	1	1	1	1
EF14	117	7799	143	*vanB*	1	1	1	1	1	1	1
EF16	117	7799	143	*vanB*	1	1	1	1	1	1	1
EF17	117	7799	143	*vanB*	1	1	1	1	1	1	1
EF18	117	7799	143	*vanB*	1	1	1	1	1	1	1
EF19	117	7799	143	*vanB*	1	1	1	1	1	1	1
EF20	117	7799	143	*vanB*	1	1	1	1	1	1	1
EF21	117	7799	143	*vanB*	1	1	1	1	1	1	1
EF22	117	7799	143	*vanB*	1	1	1	1	1	1	1
EF23	117	7799	143	*vanB*	1	1	1	1	1	1	1
EF24	117	7799	143	*vanB*	1	1	1	1	1	1	1
EF25	117	7799	143	*vanB*	1	1	1	1	1	1	1
EF26	117	7799	143	*vanB*	1	1	1	1	1	1	1
EF28	117	7799	143	*vanB*	1	1	1	1	1	1	1
EF30	117	7799	143	*vanB*	1	1	1	1	1	1	1
EF33	117	7799	143	*vanB*	1	1	1	1	1	1	1
EF35	117	7799	143	*vanB*	1	1	1	1	1	1	1
EF37	117	7799	143	*vanB*	1	1	1	1	1	1	1
EF40	117	7799	143	*vanB*	1	1	1	1	1	1	1
Local controls											
EF27	787	9254	unknown	*vanB*	1	1	1	1	1	1	1
EFK1	17	496	unknown	not found	not found	not found	not found	not found	not found	not found	not found
EFK3	117	2035	123	not found	not found	not found	not found	not found	not found	not found	not found
Reference controls											
UW20739	117	71	17	*vanB*	1	1	not found	not found	not found	1	1
UW17372	117	71	17	*vanB*	1	1	1	not found	not found	1	1
UW23490	117	71	17	*vanB*	1	1	1	not found	not found	1	2
UW23301	117	929	18	*vanB*	1	1	1	1	3	1	2
UW22604	80	1065	21	*vanB*	1	1	1	1	2	1	1
UW23773	80	1065	21	*vanB*	1	1	2	1	4	1	1
UW25395	80	2406	327	*vanB*	1	1	1	not found	not found	1	1
UW21319	1299	1903	1566	*vanA*	not found	not found	not found	not found	not found	not found	not found
Long‐term exposure											
EF12‐II	117	7799	143	*vanB*	1	1	1	2	1	1	1

*Note:* Sequence types (ST) were defined by analysis of seven housekeeping genes (Homan cite); complex types (CT) were assigned in SeqSphere (cluster threshold of ≤ 20 alleles difference to founder of complex). ST* is based on the newer MLST scheme by Bezdicek et al. (2023). Alleles were numbered sequentially to distinguish between different sequences of a gene. The table includes 20 outbreak isolates (CT7799), three local controls from the same period, eight RKI reference genomes, and one long‐term exposure replicate (EF12‐II). All isolates within the outbreak cluster share identical sequences of all genes responsible for vancomycin resistance (allele 1). One local control isolate without the specific phenotype (EF27) also carried the same alleles. The remaining local controls were susceptible to vancomycin. ST117/CT71 reference isolates were the closest genetic relatives, but each lacked multiple resistance genes. One RKI control carried *vanA*‐type resistance, but no *vanB*‐type resistance genes. The long‐term exposure replicate exhibited a variant sequence of *vanS*‐B. Van. res., type of vancomycin resistance.

### 3.2 Evaluation of Various AST Methods to Detect VREfm_styr_

**Figure 4:**
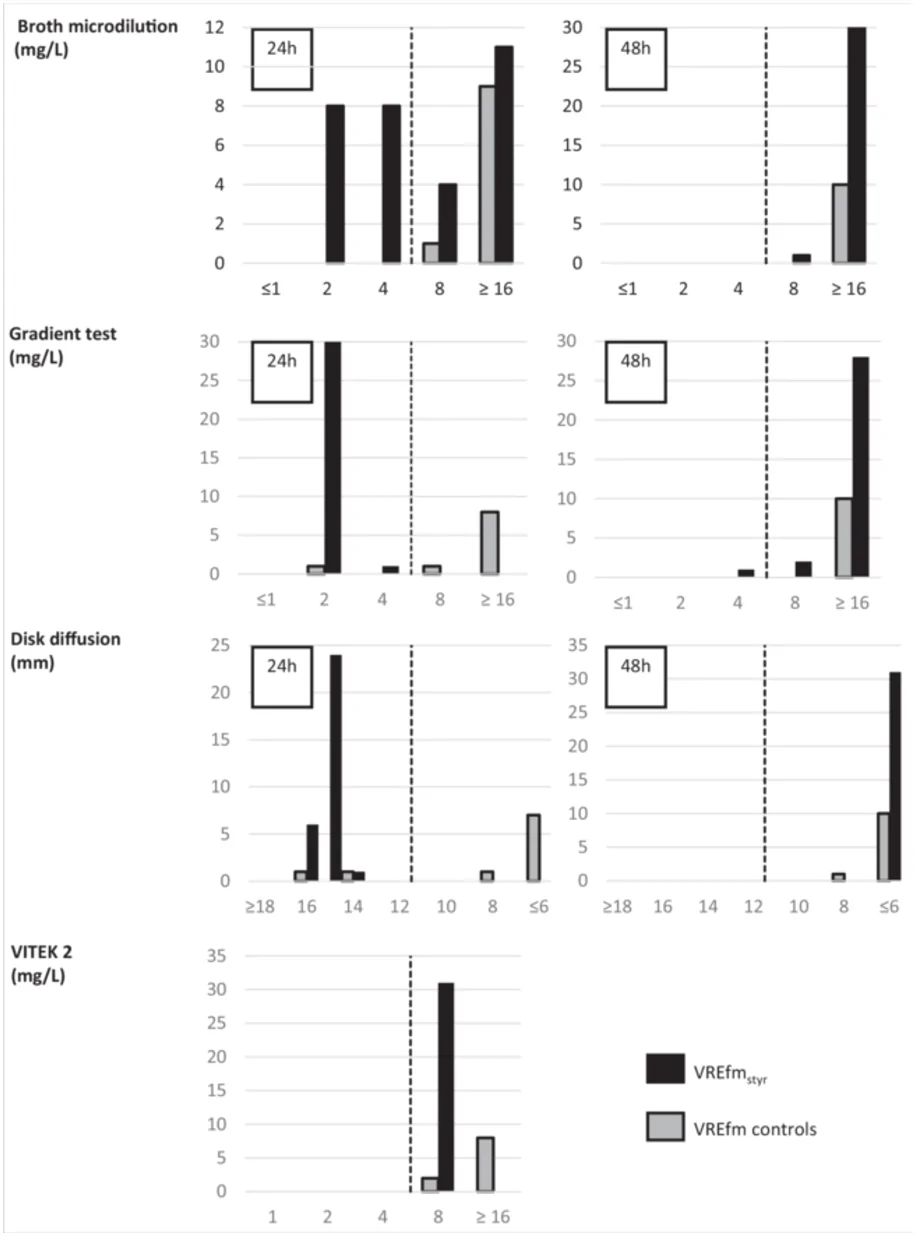
Grouped minimum inhibitory concentrations (in mg/L) and zone diameters (in mm), respectively, of 31 VREfm_styr_ outbreak isolates and ten vancomycin‐resistant *Enterococcus faecium* (VREfm) controls in different antimicrobial susceptibility testing assays after 24 and 48 h of incubation at 35°C ± 2°C, respectively. EUCAST breakpoints (*S* ≤ 4 mg/L for all MIC based analyses and *S* ≥ 12 mm for agar‐based analysis) indicated as dotted lines.

**Figure 5:**
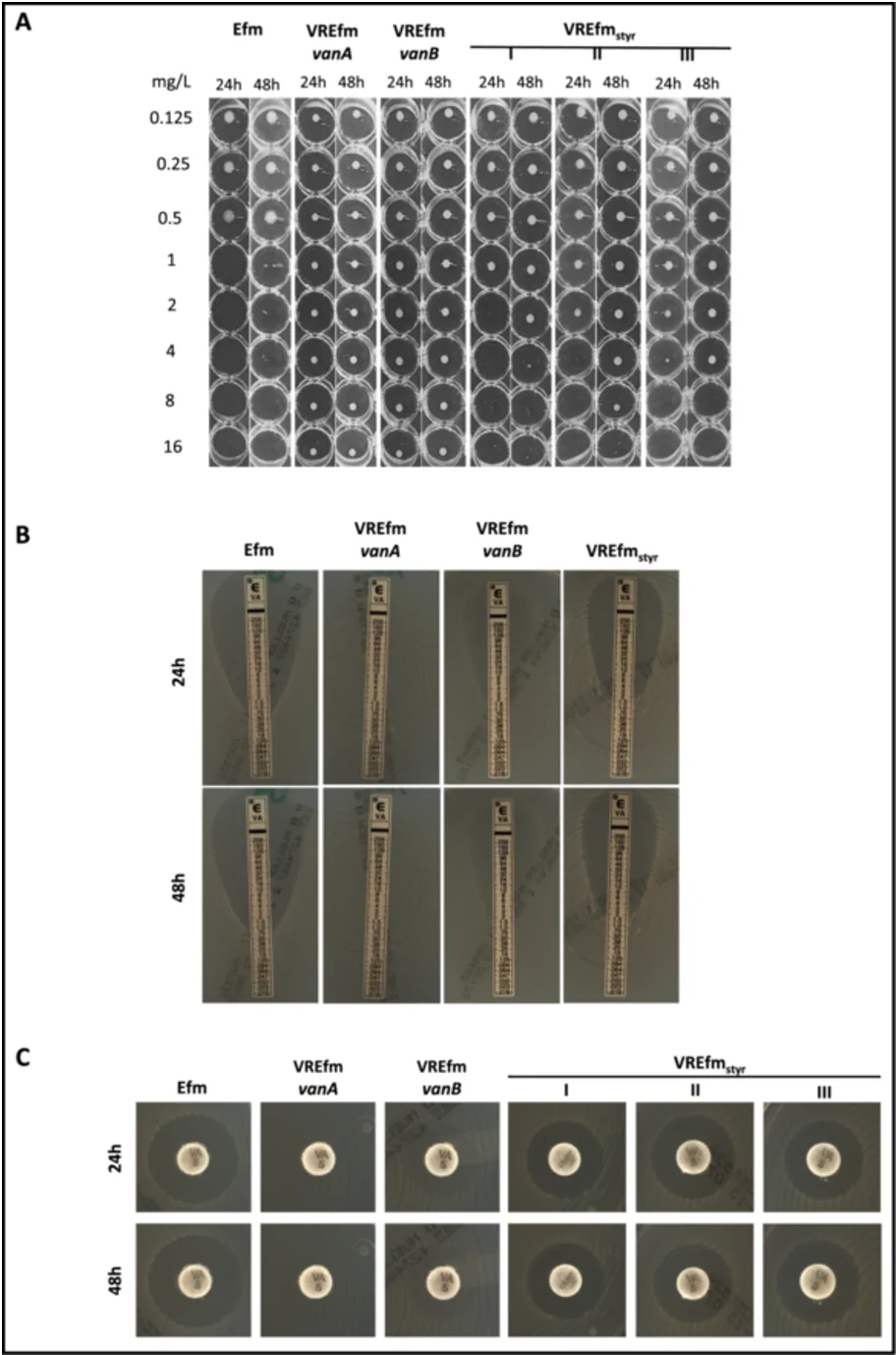
Growth phenotypes of vancomycin‐susceptible *E. faecium* strain ATCC19434 (Efm), *vanA*‐positive strain DSM17050 (VREfm *vanA*), *vanB*‐positive strain DSM25699 (VREfm *vanB*), and representative VREfm_styr_ strain(s) (I‐III). All tests were incubated at 35°C ± 2°C for 24 and 48 h. A. Isolates in vancomycin broth microdilution with three different VREfm_styr_ isolates displaying the three most frequent phenotypes: minimum inhibitory concentrations (MICs) of 2, 4, and 8 mg/L after 24 h, two strains showing further increased MICs after 48 h. B. Pictures of gradient tests (bioMérieux). C. Pictures of vancomycin disk diffusion.

When reading BMD results after 24 h of incubation, only 16 of 31 isolates (52%) were correctly identified as vancomycin resistant, whereas all included reference strains (*n* = 2) and control strains provided by the German reference center (*n* = 10; Supporting Information S2: Table S1) had minimal inhibitory concentrations (MICs) clearly > 4 mg/L (Figure 4). After 48 h, MICs of all isolates exceeded this breakpoint. However, some isolates showed exceptionally meagre growth from 2 mg/L upwards, which was easily missed even by trained personnel (Figure 5A). Similarly, GT of two different manufacturers failed to detect vancomycin resistance in all cases after 24 h of incubation (Figure 4). Most isolates (30/31; 97%) were found resistant after prolonged incubation (48 h), although, similar to BMD, growth around the gradient strip was barely visible compared to the reference strains (Figure 5B). Likewise, all isolates demonstrated disk diffusion diameters clearly wider than 11 mm after 24 h (EUCAST breakpoint for resistance: < 12 mm) (Figure 4). As vancomycin DD interpretation requires careful assessment of zone edge appearance in addition to zone diameter, it is notable that the edges of the inhibition zones did not feature the typically fuzzy appearance predictive of vancomycin resistance after 24 h (Figure 5C). After 48 h, only partial growth inhibition was visible in DD: barely visible colonies grew in the inhibition zone, which did not change in diameter (Figures 4 and 5C), matching our findings from GT plates. VITEK2 analysis with routine settings correctly identified all 31 of our isolates as VRE (Figure 4).

Upon long‐time exposure (> 100 h) of a representative VREfm_styr_ isolate (EF12) to vancomycin GT, single colonies grew in the inhibition zone. After two passages, the clone consistently presented a vancomycin MIC ≥ 32 mg/L. Sequencing data of this re‐cultivated replicate demonstrated a single base difference in the *vanS*‐B gene compared to the original isolate causing an amino acid shift (S‐243‐R) (Table 1).

#### 3.2.1 Evaluation of Various VRE Screening Agars to Detect VREfm_styr_

Kanamycin‐vancomycin (KV) agar and four different commercially available VRE screening agars (Supporting Information S2: Table S1) were inoculated with seven VREfm_styr_ isolates and additional control strains according to the manufacturers' recommendations. After 24 h, all plates showed high and clearly visible colony counts of the *vanA* VREfm control strain (Supporting Information S1: Figure S3, Table 2, Supporting Information S2: Table S4). The *vanB*‐positive VREfm control strain formed visible colonies on all but one screening agar (CHROMID‐VRE). However, three out of four screening agars failed to yield growth of VREfm_styr_ after 24 h (Brilliance‐VRE, CHROMID‐VRE, CHROMATIC‐VRE). The sole screening agar capable of growing this strain was only slightly positive for four and negative for the remaining three of the seven tested isolates (CHROMAgar). Prolonged incubation for 48 h increased the yield (Supporting Information S1: Figure S4): the initially slightly positive screening agar was now clearly positive for all tested isolates. Another screening agar yielded at least weak growth of all isolates (Brilliance‐VRE). The other two agars remained negative for the majority of VREfm_styr_ isolates, and prolonging the incubation time to 72 h only marginally improved these results (Table 2, Supporting Information S1: Figure S5). In contrast to the control strains, VREfm_styr_ failed to grow on KV agar.

**Table 2:** Growth of different VREfm strains on blood agar (BA), kanamycin‐vancomycin agar (KV), and commercial VRE screening media (CHR, CHROMagar VRE; OXO, Brilliance VRE; LIO, CHROMID VRE; BMX, chromID VRE).

	Strain	BA	CHR	OXO	LIO	BMX	KV
24 h	VREfm *vanA*	+	+	+	+	+	+
	VREfm *vanB*	+	+	±	±	—	—
	VREfm_styr_	+	+	—	—	—	—
48 h	VREfm *vanA*	+	+	+	+	+	+
	VREfm *vanB*	+	+	+	+	±	±
	VREfm_styr_	+	+	±	—	—	—
72 h	VREfm *van*A	+	+	+	+	+	+
	VREfm *vanB*	+	+	+	+	+	±
	VREfm_styr_	+	+	+	—	±	—

*Note:* Results shown are based on seven randomly selected non‐duplicate isolates of VREfm_styr_. Growth patterns represent the majority outcome per medium. +, confluent growth of colonies; ±, growth of single colonies; —, no growth.

## 4 Discussion

Our study shows that South‐eastern Austria is experiencing a sharp increase of VREfm cases, due to the newly emerged and yet unreported strain ST117/CT7799 (Abdelbary et al. 2019; Caplunik‐Pratsch et al. 2024). For ST117, *vanB*‐type vancomycin resistance has just recently been described (Brajerova et al. 2025). The outbreak characteristics suggest that transmission occurred within the hospital environment (Bender et al. 2022). Particularly, the disproportionate rise in invasive infections is a cause for concern—especially since the unique growth characteristics and resistance phenotype make it very plausible that VREfm_styr_ would not be detected in many routine settings, since many laboratories rely on 24 h DD zone diameter readings to determine susceptibility. The strains in our study were most likely transmitted in the hospital setting and displayed a remarkable genotypic stability although cultivated many months apart.

Molecular typing revealed the complete sequence of the transposon Tn*1549* carrying the genes conferring vancomycin resistance (Grabsch et al. 2008). The reference sequence was, however, interrupted by insertion elements, which were only identifiable by LR assembly. The comparison to other VRE (either reference or RKI strains) revealed single base differences in *vanR*‐B, the carboxypeptidase coding *vanY*‐B and the sequence coding for the accessory protein VanW‐B of VREfm_styr_. Whether these mutations or the genetic arrangement are causative to the resistance pattern, should be further investigated.

There are only few reports on VRE displaying low vancomycin MICs (LM‐VRE), particularly vancomycin variable enterococci (VVE) of the *vanA* genotype and VREfm positive for the *vanB*2‐Tn*5382* cluster type (Grabsch et al. 2008; Werner et al. 2012; Hammerum et al. 2017; Szakacs et al. 2014; Klare et al. 2012). A previous study reported a low sensitivity of GT, which—according to the 2018 EUCAST warning—may even fail to detect *vanB*‐mediated resistance (Klare et al. 2019; The European Committee on Antimicrobial Susceptibility Testing 2021). However, none of the conventional methods was found to be sufficient for detecting VREfm_styr_: in DD, no isolate displayed zone diameters < 12 mm, gradient testing was completely unable to detect vancomycin resistance, and even BMD, representing the gold standard, only identified 16/31 (52%) of the tested isolates after the standard incubation time. While prolonged incubation significantly improved sensitivity, this approach will alter the routines of diagnostic laboratories and delay the reporting of AST results for the vast majority of Efm isolates, that is, non‐VRE strains. Contrarily, VITEK2 analysis was found to be a helpful tool for the detection of VREfm_styr_, since it was the only phenotypic assay to identify all specific strains under routine conditions. This observation diverges from previous studies, reporting a sensitivity of only 81% in LM‐VRE (Klare et al. 2019). This result, however, was obtained in 2019 using AST card P611, hence, it is conceivable that subsequent card revisions and software updates have enhanced the system's performance (Klare et al. 2019).

There are neither specific reports about Efm ST117/CT7799 in the literature nor any warnings about vancomycin resistant strains of this lineage. Combined with the absence of this phenotype in our laboratory records before 2024, the findings indicate that the described VREfm strain has only recently emerged. Non‐selective screening methods are likely insufficient to detect VRE, as it often represents only a small fraction of the intestinal enterococcal population (Werner et al. 2012). While enterococci with high‐level vancomycin resistance can be detected reliably with chromogenic screening media, sensitivity significantly decreases for VRE isolates displaying MICs < 16 mg/L (Klare et al. 2012; Wijesuriya et al. 2014; Adler et al. 2010). Screening becomes particularly challenging when VVEs are involved (Wijesuriya et al. 2014). For the detection of VREfm_styr_, CHROMagar VRE was the only reliable screening agar after 24 h incubation. The manufacturers of all other tested media generally suggest prolonged incubation, following the recommendation of previous comparative evaluations (Adler et al. 2010; Boschert et al. 2023). However, prolonged incubation was still not sufficient for reliable detection of VREfm_styr_, and the results varied greatly between manufacturers. This adaptation also has further limitations: increasing the standard time of analysis to two or even 3 days delays efficient outbreak management and forces the healthcare centers to isolate patients at‐risk with unknown VRE status for additional days, which will increase costs.

## 5 Conclusions

This study provides extensive insight into an emerging, yet undescribed VREfm strain, including aspects of infection control and diagnostic microbiology, for example, the presumed superiority of VITEK2 testing. However, the presented findings might lead to overestimate this assay's sensitivity: with VITEK2 positivity being the inclusion criterion for this study, it is conceivable that a VREfm_styr_ subpopulation overseen by VITEK2 might have been misdiagnosed in our laboratory.

The observed emergence of VREfm_styr_ in South‐Eastern Austria not only underscores its epidemic potential, but also reveals a serious diagnostic gap, as current screening strategies and conventional AST‐methods fail to reliably detect these cases. Our findings suggest that laboratories 1) must verify that their screening media and incubation time are sufficient and 2) need to adapt their AST protocols, that is, prolong incubation, involve molecular *vanB*‐testing, or apply VITEK2 testing, which proved to be the superior method for identification of VREfm_styr_.

Further research is required to elucidate the mechanisms causing this (primarily) occult VRE phenotype. Surveillance studies are necessary to monitor the epidemiology of VREfm_styr_ in order to investigate whether its emergence is a local phenomenon—or whether laboratories in other regions need to adapt their diagnostic procedures as well.

## Author Contributions

**Patrick Forstner:** conceptualization (equal), methodology (equal), formal analysis (equal), investigation (lead), writing – original draft (equal), writing – review and editing (equal), visualization (equal). **Christine Uitz:** conceptualization (equal), methodology (equal), formal analysis (equal), investigation (lead), writing – original draft (equal), writing – review and editing (equal), visualization (equal). **Johanna Dabernig‐Heinz:** methodology (equal), formal analysis (equal), investigation (lead), writing – review and editing (equal), visualization (equal). **Gabriel E. Wagner:** methodology (equal), investigation (lead), writing – review and editing (equal), visualization (support). **Jennifer Bender:** investigation (supporting), writing – review and editing (equal). **Martin Fischer:** investigation (supporting), writing – review and editing (equal). **David Siebenhofer:** investigation (supporting), writing – review and editing (equal). **Guido Werner:** investigation (supporting), resources (supporting), writing – review and editing (equal). **Tobias Busche:** investigation (supporting), resources (supporting), writing – review and editing (equal). **Levin Joe Klages** investigation (supporting), writing – review and editing (equal). **Christian Rückert‐Reed:** methodology (equal), investigation (lead), writing – review and editing (equal), visualization (support). **Ivo Steinmetz:** resources (lead), writing – review and editing (equal), supervision (equal). **Karl Dichtl:** conceptualization (equal), methodology (equal), formal analysis (equal), investigation (lead), resources (supporting), writing – original draft (equal), writing – review and editing (equal), visualization (equal), supervision (equal), project administration (lead).

## Funding

The authors have nothing to report.

## Ethics Statement

The study protocol was approved by the ethics committee of the Medical University of Graz (#35‐401) and conducted in accordance with the declaration of Helsinki. A waiver of informed consent was granted.

## Conflicts of Interest

K.D. received technical support and consumables by bioMérieux, unrelated to the present study. D.S. reports travel support and honoraria for a presentation and an on‐site visit related to an educational event. P.F., C.U., J.D.H., G.E.W., J.B., M.F., G.W., T.B., L.K., C.R.R. and I.S. declare no conflicts of interest.

## Declaration of Generative AI and AI‐Assisted Technologies

During manuscript preparation, the authors used DeepL and Grammarly in order to check grammar and spelling to improve readability. All content was critically reviewed and edited by the authors, who take full responsibility for its accuracy and integrity.

## Supporting information


Supporting File 1

Supporting File 2

## Data Availability

Sequencing data was deposited at the SRA BioProject Number PRJNA1359736. Other data are available from the authors on reasonable request.

## References

[mbo370393-bib-0001] Abdelbary, M. H. H. , L. Senn , G. Greub , G. Chaillou , E. Moulin , and D. S. Blanc . 2019. “Whole‐Genome Sequencing Revealed Independent Emergence of Vancomycin‐Resistant Enterococcus faecium Causing Sequential Outbreaks Over 3 Years in a Tertiary Care Hospital.” European Journal of Clinical Microbiology & Infectious Diseases: Official Publication of the European Society of Clinical Microbiology 38, no. 6 (June): 1163–1170. 10.1007/s10096-019-03524-z. 30888549

[mbo370393-bib-0002] Adler, H. , S. Oezcan , and R. Frei . 2010. “Vancomycin‐Resistant Enterococci of Vanb Genotype May Pose Problems for Screening With Highly Selective Media.” Journal of Clinical Microbiology 48, no. 6 (June): 2323. 10.1128/JCM.00631-10. 20410340 PMC2884490

[mbo370393-bib-0003] Al Rubaye, M. , J. Janice , J. V. Bjørnholt , et al. 2023. “The Population Structure of Vancomycin‐Resistant and ‐Susceptible Enterococcus faecium in a Low‐Prevalence Antimicrobial Resistance Setting Is Highly Influenced by Circulating Global Hospital‐Associated Clones.” Microbial Genomics 9, no. 12 (December): 001160. 10.1099/mgen.0.001160. 38112685 PMC10763505

[mbo370393-bib-0004] de Been, M. , M. Pinholt , J. Top , et al. 2015. “Core Genome Multilocus Sequence Typing Scheme for High‐ Resolution Typing of Enterococcus Faecium.” Journal of Clinical Microbiology 53, no. 12 (December): 3788–3797. 10.1128/JCM.01946-15. 26400782 PMC4652124

[mbo370393-bib-0005] Bender, J. K. , J. Hermes , L. T. Zabel , et al. 2022. “Controlling an Unprecedented Outbreak With Vancomycin‐Resistant Enterococcus faecium in Germany, October 2015 to November 2019.” Microorganisms 10, no. 8 (August): 1603. 10.3390/microorganisms10081603. 36014021 PMC9412439

[mbo370393-bib-0006] Bezdicek, M. , J. Hanslikova , M. Nykrynova , et al. 2023. “New Multilocus Sequence Typing Scheme for Enterococcus faecium Based on Whole Genome Sequencing Data.” Microbiology Spectrum 11, no. 4 (August): e05107‐22. 10.1128/spectrum.05107-22. 37306567 PMC10434285

[mbo370393-bib-0007] Blaskovich, M. A. T. , K. A. Hansford , M. S. Butler , Z. Jia , A. E. Mark , and M. A. Cooper . 2018. “Developments in Glycopeptide Antibiotics.” ACS Infectious Diseases 4, no. 5 (May): 715–735. 10.1021/acsinfecdis.7b00258. 29363950 PMC5952257

[mbo370393-bib-0008] Boschert, A. L. , F. Arndt , A. Hamprecht , M. Wolke , and S. V. Walker . 2023. “Comparison of Five Different Selective Agar for the Detection of Vancomycin‐Resistant *Enterococcus faecium* .” Antibiotics (Basel, Switzerland) 12, no. 4 (March): 666. 10.3390/antibiotics12040666. 37107028 PMC10135216

[mbo370393-bib-0009] Brajerova, M. , O. Nyc , P. Drevinek , and M. Krutova . 2025. “Genomic Insights Into the Spread of Vancomycin‐ and Tigecycline‐Resistant Enterococcus faecium St117.” Annals of Clinical Microbiology and Antimicrobials 24, no. 1 (June): 36. 10.1186/s12941-025-00806-7. 40500694 PMC12153105

[mbo370393-bib-0010] Bundesministerium für Soziales GP und K (BMSGPK) . 2024. Resistenzbericht Österreich – AURES 2023.

[mbo370393-bib-0011] Caplunik‐Pratsch, A. , B. Kieninger , V. A. Donauer , et al. 2024. “Introduction and Spread of Vancomycin‐Resistant Enterococcus Faecium (VREfm) at a German Tertiary Care Medical Center From 2004 Until 2010: A Retrospective Whole‐Genome Sequencing (WGS) Study of the Molecular Epidemiology of Vrefm.” Antimicrobial Resistance & Infection Control 13, no. 1 (February): 20. 10.1186/s13756-024-01379-4. 38355509 PMC10865517

[mbo370393-bib-0012] Cetinkaya, Y. , P. Falk , and C. G. Mayhall . 2000. “Vancomycin‐Resistant Enterococci.” Clinical Microbiology Reviews 13, no. 4 (October): 686–707. 10.1128/CMR.13.4.686. 11023964 PMC88957

[mbo370393-bib-0013] Dabernig‐Heinz, J. , M. Lohde , M. Hölzer , et al. 2024. “A Multicenter Study on Accuracy and Reproducibility of Nanopore Sequencing‐Based Genotyping of Bacterial Pathogens.” Journal of Clinical Microbiology 62, no. 9 (September): e0062824. 10.1128/jcm.00628-24. 39158309 PMC11389150

[mbo370393-bib-0014] European Centre for Disease Prevention and Control . 2024. Antimicrobial Resistance in the EU/EEA (EARS‐Net) ‐ Annual Epidemiological Report 2023. Stockholm.

[mbo370393-bib-0015] Feldgarden, M. , V. Brover , N. Gonzalez‐Escalona , et al. 2021. “Amrfinderplus and the Reference Gene Catalog Facilitate Examination of the Genomic Links Among Antimicrobial Resistance, Stress Response, and Virulence.” Scientific Reports 11, no. 1 (June): 12728. 10.1038/s41598-021-91456-0. 34135355 PMC8208984

[mbo370393-bib-0016] Goig, G. A. , S. Blanco , A. L. Garcia‐Basteiro , and I. Comas . 2020. “Contaminant DNA in Bacterial Sequencing Experiments Is a Major Source of False Genetic Variability.” BMC Biology 18, no. 1 (March): 24. 10.1186/S12915-020-0748-Z. 32122347 PMC7053099

[mbo370393-bib-0017] Grabsch, E. A. , K. Chua , S. Xie , et al. 2008. “Improved Detection of vanB2‐containing Enterococcus Faecium With Vancomycin Susceptibility by Etest Using Oxgall Supplementation.” Journal of Clinical Microbiology 46, no. 6 (June): 1961–1964. 10.1128/JCM.01778-07. 18434564 PMC2446868

[mbo370393-bib-0018] Hammerum, A. M. , S. Baig , Y. Kamel , et al. 2017. “Emergence of vanA Enterococcus faecium in Denmark, 2005–15.” Journal of Antimicrobial Chemotherapy 72, no. 8 (August): 2184–2190. 10.1093/jac/dkx138. 28541565

[mbo370393-bib-0019] Klare, I. , J. K. Bender , C. Fleige , et al. 2019. “Comparison of VITEK® 2, Three Different Gradient Strip Tests and Broth Microdilution for Detecting Vanb‐Positive Enterococcus Faecium Isolates With Low Vancomycin MICs.” Journal of Antimicrobial Chemotherapy 74, no. 10 (October): 2926–2929. 10.1093/jac/dkz310. 31335935 PMC6753474

[mbo370393-bib-0020] Klare, I. , C. Fleige , U. Geringer , W. Witte , and G. Werner . 2012. “Performance of Three Chromogenic Vre Screening Agars, Two Etest(®) Vancomycin Protocols, and Different Microdilution Methods in Detecting Vanb Genotype Enterococcus faecium With Varying Vancomycin MICs.” Diagnostic Microbiology and Infectious Disease 74, no. 2 (October): 171–176. 10.1016/j.diagmicrobio.2012.06.020. 22901792

[mbo370393-bib-0021] Knudsen, M. J. S. , J. A. S. Castruita , I. M. C. Rubin , et al. 2025. “Genomic Epidemiology of Vancomycin‐Resistant Enterococcus Faecium in Eastern Denmark From 2020 to 2022, and Identification of Vanb Tn1549 Insertion Sites.” European Journal of Clinical Microbiology & Infectious Diseases 44, no. 6 (June): 1425–1432. 10.1007/s10096-025-05091-y. 40167959 PMC12116600

[mbo370393-bib-0022] Leclercq, R. , E. Derlot , J. Duval , and P. Courvalin . 1988. “Plasmid‐Mediated Resistance to Vancomycin and Teicoplanin in Enterococcus faecium.” New England Journal of Medicine 319, no. 3 (July): 157–161. 10.1056/NEJM198807213190307. 2968517

[mbo370393-bib-0023] McCormick, M. H. , J. M. McGuire , G. E. Pittenger , R. C. Pittenger , and W. M. Stark . 1955. “Vancomycin, a New Antibiotic. I. Chemical and Biologic Properties.” Antibiotics Annual 3: 606–611. 13355336

[mbo370393-bib-0024] miGenomeSurv‐Consortium . https://www.medizin.uni-muenster.de/migenomesurv/home.html. 2021. Microbial Genomic Surveillance (miGenomeSurv).

[mbo370393-bib-0025] Prematunge, C. , C. MacDougall , J. Johnstone , et al. 2016. “VRE and VSE Bacteremia Outcomes in the Era of Effective VRE Therapy: A Systematic Review and Meta‐Analysis.” Infection Control & Hospital Epidemiology 37, no. 1 (January): 26–35. 10.1017/ice.2015.228. 26434609 PMC4707508

[mbo370393-bib-0026] Souvorov, A. , R. Agarwala , and D. J. Lipman . 2018. “SKESA: Strategic K‐Mer Extension for Scrupulous Assemblies.” Genome Biology 19, no. 1 (October): 153. 10.1186/s13059-018-1540-z. 30286803 PMC6172800

[mbo370393-bib-0027] Szakacs, T. A. , L. Kalan , M. J. McConnell , et al. 2014. “Outbreak of Vancomycin‐Susceptible Enterococcus faecium Containing the Wild‐Type vanA Gene.” Journal of Clinical Microbiology 52, no. 5 (May): 1682–1686. 10.1128/JCM.03563-13. 24523464 PMC3993680

[mbo370393-bib-0028] The European Committee on Antimicrobial Susceptibility Testing . 2021. “Vancomycin susceptibility testing in Enterococcus faecalis and E.” In Faecium Using MIC Gradient Tests. Stockholm.

[mbo370393-bib-0029] The European Committee on Antimicrobial Susceptibility Testing . 2025. Breakpoint Tables for Interpretation of MICs and Zone Diameters. Version 15.0 [Internet]. 15.0. https://www.eucast.org/bacteria/document-archive/.

[mbo370393-bib-0030] Uttley, A. C. , C. H. Collins , J. Naidoo , and R. C. George . 1988. “Vancomycin‐Resistant Enterococci.” Lancet 331, no. 8575–6: 57–58. 10.1016/s0140-6736(88)91037-9. 2891921

[mbo370393-bib-0031] Wagner, G. E. , J. Dabernig‐Heinz , M. Lipp , et al. 2023. “Real‐Time Nanopore Q20+ Sequencing Enables Extremely Fast and Accurate Core Genome MLST Typing and Democratizes Access to High‐Resolution Bacterial Pathogen Surveillance.” Journal of Clinical Microbiology 61, no. 4 (April): e0163122. 10.1128/jcm.01631-22. 36988494 PMC10117118

[mbo370393-bib-0032] Werner, G. , I. Klare , C. Fleige , et al. 2012. “Vancomycin‐Resistant Vanb‐Type Enterococcus faecium Isolates Expressing Varying Levels of Vancomycin Resistance and Being Highly Prevalent Among Neonatal Patients in a Single ICU.” Antimicrobial Resistance and Infection Control 1, no. 1 (May): 21. 10.1186/2047-2994-1-21. 22958440 PMC3533821

[mbo370393-bib-0033] Wijesuriya, T. M. , P. Perry , T. Pryce , et al. 2014. “Low Vancomycin MICs and Fecal Densities Reduce the Sensitivity of Screening Methods for Vancomycin Resistance in Enterococci.” Journal of Clinical Microbiology 52, no. 8 (August): 2829–2833. 10.1128/JCM.00021-14. 24871216 PMC4136124

[mbo370393-bib-0034] World Health Organization . 2024. WHO Bacterial Priority Pathogens List 2024: Bacterial Pathogens of Public Health Importance, to Guide Research, Development, and Strategies to Prevent and Control Antimicrobial Resistance. World Health Organization.

