# Supplementary material for: Emergence of a Novel, Phenotypically Difficult‐to‐Detect Vancomycin‐Resistant Enterococcus faecium Clone (ST117/CT7799)
Source: Microbiologyopen. 2026 Aug 31;15(5):e70393. doi: 10.1002/mbo3.70393 (PMC13528232; doi:10.1002/mbo3.70393)
Supplement: Supplementary file 1 [file MBO3-15-e70393-s001.docx]

| **Product name** | **Manufacturer** | **Ref number** |
| --- | --- | --- |
| **Media** |  |  |
| Columbia blood Agar | Becton Dickinson, Franklin Lakes, USA | 254071 |
| Mueller Hinton II Agar | Becton Dickinson, Franklin Lakes, USA | 254081 |
| CHROMagar VRE | CHROMagar, Paris, France | 201460 |
| CHROMID VRE agar | bioMérieux, Marcy-L’Étoile, France | 43004 |
| Chromatic VRE | Liofilchem, Teramo, Italy | 11621 |
| Brilliance VRE Agar | Oxoid, Hampshire, England | PO1175A |
| Schaedler Kanamycin-Vancomycin Agar | Becton Dickinson, Franklin Lake, USA, | 254077 |
| **AST systems and supplies** |  |  |
| Broth microdilution |  |  |
| Sensititre Susceptibility Plate YEUENCF | TREK Diagnostic Systems, East Grinstead, UK | YT3462 |
| Sensititre Mueller Hinton broth | TREK Diagnostic Systems, East Grinstead, UK | YEUENCF |
| Disk diffusion |  |  |
| MASTDISCS Vancomycin VA5 | Mast Group Ltd., Merseyside, UK | 112027 |
| Vancomycin 5 µg discs | Becton Dickinson, Franklin Lake, USA | 231352 |
| Gradient testing |  |  |
| ETEST Vancomycin | bioMérieux, Marcy-L’Étoile, France | 412488 |
| MIC Test Strip Vancomycin | Liofilchem, Roseto degli Abruzzi, Italy | 92057 |
| VITEK 2 testing |  |  |
| VITEK 2 XL | bioMérieux, Marcy-L’Étoile, France | SYS314 |
| AST-P655 | bioMérieux, Marcy-L’Étoile, France | 421913 |
| **WGS and molecular analysis** |  |  |
| GeneXpert Dx IV | Cepheid AB, Solna, Sweden | GX4-CE-IV |
| Xpert *vanA*/*vanB* | Cepheid AB, Solna, Sweden | GXVANA/B-CE-10 |
| NucleoSpin Microbial DNA Kit | Macherey-Nagel, Düren, Deutschland |  |
| AMPure XP Isolation kit | Beckman Coulter, Brea, USA |  |
| Native barcoding kit (SQK-NBD114.24) | Oxford Nanopore Technologies, Oxford, UK |  |
| MinION Mk1C | Oxford Nanopore Technologies, Oxford, UK |  |
| R.10.4.1 flow cell | Oxford Nanopore Technologies, Oxford, UK |  |
| Flye v2.9.5 | https://github.com/mikolmogorov/Flye |  |
| Medaka v2.0.1 | https://github.com/nanoporetech/medaka | Model: dna_r10.4.1_e8.2_400bps_sup@v5.0#.0 |
| Nextera XT preparation kit | Illumina, San Diego, USA |  |
| Nextseq200 with P1 cartridge | Illumina, San Diego, USA |  |
| SKESA, v2.3.0 | https://github.com/ncbi/SKESA |  |
| SeqSphere+ v10.0.4 | Ridom GmbH, Münster, Germany |  |
| Blastn v2.9.0 | National Center for Biotechnology Information, Maryland, USA |  |
| Tn*1549* sequence | - | ncbi.nlm.nih.gov/nuccore/AF192329 |
| **Laboratory devices** |  |  |
| Kiestra laboratory automation system | Becton Dickinson, Franklin Lakes, USA |  |
| MALDI Biotyper sirius | Bruker Daltonics, Bremen, Germany | 1890111 |
| MBT IVD Library Revision G (Claim 6) | Bruker Daltonics, Bremen, Germany | 1846904 |
| **Control strains** |  |  |
| ATCC19434 (vancomycin-susceptible) | American Type Culture Collection, Manassas, USA | ATCC19434 |
| *Enterococcus faecium* VRE (*vanA*) | DSMZ, Braunschweig, Germany | DSM1705 |
| *Enterococcus faecium* VRE (*vanB*) | DSMZ, Braunschweig, Germany | DSM25699 |
| *Enterococcus faecium* VRE patient isolates | Robert Koch Institute, Wernigerode, Germany |  |
| ST1299/CT1903 *vanA* | Robert Koch Institute, Wernigerode, Germany | UW21319 |
| ST117/CT71 *vanB* | Robert Koch Institute, Wernigerode, Germany | UW17372 |
| ST117/CT71 *vanB* | Robert Koch Institute, Wernigerode, Germany | UW20739 |
| ST80/CT1065 *vanB* | Robert Koch Institute, Wernigerode, Germany | UW22604 |
| ST117/CT929 *vanB* | Robert Koch Institute, Wernigerode, Germany | UW23301 |
| ST80/CT1065 *vanB* | Robert Koch Institute, Wernigerode, Germany | UW23773 |
| ST80/CT2406 *vanB* | Robert Koch Institute, Wernigerode, Germany | UW25395 |
| ST117/CT71 *vanB* | Robert Koch Institute, Wernigerode, Germany | UW23490 |

**Supplementary Table S1.** Media, antimicrobial susceptibility testing (AST) systems and reagents, consumables, laboratory instrumentation, and control strains. DSMZ, German Collection of Microorganisms and Cell Cultures.

| **Case** | **Age** | **Specimen** | **Discipline(s)** | **Hospitalization** | **History of inpatient care within last 6 months** |
| --- | --- | --- | --- | --- | --- |
| EF12 | 66 | blood culture | general surgery | Yes | - |
| EF13 | 42 | blood culture | general surgery | Yes | - |
| EF14 | 79 | blood culture | general surgery | Yes | - |
| EF16 | 35 | resp. sample | neurosurgery | Yes | - |
| EF18 | 52 | wound swab | general surgery | Yes | - |
| EF19 | 86 | wound swab | general surgery | Yes | - |
| EF20 | 67 | urine | general surgery | Yes | - |
| EF21 | 57 | wound swab | general surgery | Yes | - |
| EF22 | 66 | fluid aspirate | general surgery | Yes | - |
| EF23 | 76 | urine | general surgery | Yes | - |
| EF24 | 87 | wound swab | general surgery | Yes | - |
| EF25 | 76 | urine | general surgery, urology | No | Yes |
| EF26 | 77 | urine | general practitioner | No | No |
| EF28 | 94 | urine | urology | No | Yes |
| EF29 | 51 | urine | urology | No | Yes |
| EF30 | 74 | blood culture | general surgery | Yes | - |
| EF31 | 76 | wound swab | orth. & traum. | Yes | - |
| EF33 | 79 | urine | general practitioner | No | Yes |
| EF34 | 57 | urine | general surgery, orth. & traum. | No | Yes |
| EF35 | 85 | urine | urology | No | Yes |
| EF36 | 48 | fluid aspirate | general surgery | No | Yes |
| EF37 | 88 | urine | external hospital (int. medicine) | Yes | - |
| EF38 | 84 | urine | external hospital (int. medicine) | Yes | - |
| EF39 | 90 | urine | external hospital (int. medicine) | Yes | - |
| EF40 | 89 | blood culture | general surgery, orth. & traum. | Yes | - |
| EF41 | 56 | urine | neurology | Yes | - |
| EF42 | 81 | urine | urology | No | Yes |
| EF44 | 63 | fluid aspirate | general surgery | No | Yes |
| EF45 | 55 | wound swab | general surgery | Yes | - |
| EF46 | 84 | urine | general surgery | No | Yes |
| EF47 | 67 | fluid aspirate | urology, general surgery | Yes | - |

**Supplementary Table S2.** Characteristics of the patients tested positive for VREfm_styr_ including the clinical discipline at the time of specimen collection. Hospitalization refers to in hospital stay ≤ 4 days at the time of positive specimen collection. The history of inpatient care within last six months is only indicated for patients who were not hospitalized at the time of positive specimen collection. resp. sample, respiratory sample; orth. & traum., orthopaedics and traumatology; int. medicine, internal medicine.

| **Case** | **Data** | **Sample ID** | **number of input bases** | **read N50** | **average coverage of assembly** | **assembly length** | **contigs in final assembly** | **assembly N50** |
| --- | --- | --- | --- | --- | --- | --- | --- | --- |
| EF12 | SR | EF-VRE12-25_SR | 336.679.854 | 147 | 115 | 2.875.176 | 213 | 34.423 |
| EF12 | LR | EF12_SQK-NBD114-24_barcode04 | 1.541.919.889 | 10.385 | 508 | 3.055.308 | 5 | 2.850.443 |
| EF12-II | SR | EF-VRE12II-25_SR | 477.978.463 | 146 | 166 | 2.867.181 | 236 | 32.961 |
| EF12-II | LR | EF12II_SQK-NBD114-24_barcode05 | 1.581.576.500 | 10.794 | 511 | 3.058.264 | 5 | 2.850.445 |
| EF13 | SR | EF-VRE13-25_SR | 306.963.048 | 147 | 102 | 2.964.569 | 208 | 34.961 |
| EF13 | LR | EF13_SQK-NBD114-24_barcode06 | 1.354.395.448 | 12.023 | 427 | 3.060.898 | 5 | 2.850.445 |
| EF14 | SR | EF-VRE14-25_SR | 347.267.107 | 147 | 120 | 2.850.019 | 272 | 25.358 |
| EF14 | LR | EF14_SQK-NBD114-24_barcode07 | 1.101.178.499 | 12.036 | 356 | 3.057.683 | 5 | 2.853.832 |
| EF16 | SR | EF-VRE16-25-WH_SR | 392.506.335 | 126 | 137 | 2.874.303 | 233 | 34.005 |
| EF16 | LR | EF16_SQK-NBD114-24_barcode08 | 1.012.836.052 | 12.584 | 319 | 3.057.030 | 4 | 2.856.864 |
| EF18 | SR | EF-VRE18-25_SR | 385.291.337 | 146 | 129 | 2.970.766 | 328 | 22.212 |
| EF18 | LR | EF18_SQK-NBD114-24_barcode10 | 704.902.477 | 8.503 | 187 | 3.237.514 | 8 | 2.887.639 |
| EF19 | SR | EF-VRE19-25_SR | 373.814.441 | 147 | 128 | 2.863.079 | 251 | 29.295 |
| EF19 | LR | EF19_SQK-NBD114-24_barcode11 | 990.293.490 | 12.885 | 314 | 3.058.296 | 4 | 2.851.963 |
| EF20 | SR | EF-VRE20-25_SR | 310.902.084 | 145 | 107 | 2.869.642 | 230 | 31.261 |
| EF20 | LR | EF20_SQK-NBD114-24_barcode12 | 864.806.117 | 11.161 | 283 | 3.070.617 | 6 | 2.853.846 |
| EF21 | SR | EF-VRE21-25_SR | 308.791.849 | 147 | 105 | 2.873.767 | 217 | 33.263 |
| EF21 | LR | EF21_SQK-NBD114-24_barcode13 | 864.938.418 | 12.054 | 281 | 3.121.170 | 6 | 2.873.280 |
| EF22 | SR | EF-VRE22-25_SR | 442.111.408 | 144 | 151 | 2.875.240 | 214 | 34.424 |
| EF22 | LR | EF22_SQK-NBD114-24_barcode14 | 1.110.362.084 | 11.704 | 363 | 3.067.818 | 7 | 2.850.439 |
| EF23 | SR | EF-VRE23-25_SR | 435.670.996 | 144 | 149 | 2.874.723 | 222 | 33.263 |
| EF23 | LR | EF23_SQK-NBD114-24_barcode15 | 672.792.740 | 11.296 | 219 | 3.033.470 | 4 | 2.855.357 |
| EF24 | SR | EF-VRE24-25_SR | 473.357.742 | 146 | 163 | 2.865.290 | 245 | 27.479 |
| EF24 | LR | EF24_SQK-NBD114-24_barcode16 | 229.543.900 | 10.733 | 75 | 3.069.804 | 5 | 2.857.051 |
| EF25 | SR | EF-VRE25-25_SR | 366.277.616 | 146 | 126 | 2.857.613 | 271 | 26.746 |
| EF25 | LR | EF25_SQK-NBD114-24_barcode17 | 72.008.770 | 10.077 | 219 | 3.083.440 | 5 | 2.900.566 |
| EF26 | SR | EF-VRE26-25_SR | 426.527.907 | 145 | 148 | 2.850.126 | 289 | 25.762 |
| EF26 | LR | EF26_SQK-NBD114-24_barcode18 | 586.973.096 | 11.023 | 189 | 3.059.595 | 5 | 2.850.304 |
| EF28 | SR | EF-VRE28-25_SR | 343.757.037 | 146 | 116 | 2.902.661 | 240 | 30.211 |
| EF28 | LR | EF28_SQK-NBD114-24_barcode20 | 392.107.399 | 9.729 | 127 | 3.108.235 | 6 | 2.892.460 |
| EF30 | SR | EF30_10m_S57 | 396.140.140 | 147 | 139 | 2.821.662 | 332 | 21.226 |
| EF30 | LR | VREf30-barcode03 | 584.000.548 | 8.878 | 158 | 3.066.959 | 10 | 1.931.125 |
| EF33 | SR | EF33_10m_S58 | 373.623.805 | 147 | 125 | 2.947.845 | 316 | 24.418 |
| EF33 | LR | VREf33-barcode04 | 279.577.668 | 9.291 | 60 | 3.177.992 | 10 | 2.851.872 |
| EF35 | SR | EF35_10m_S59 | 295.203.614 | 146 | 100 | 2.904.526 | 382 | 27.362 |
| EF35 | LR | VREf35-barcode05 | 482.662.224 | 9.371 | 116 | 3.309.926 | 10 | 2.925.694 |
| EF37 | SR | EF37_10m_S60 | 353.799.878 | 145 | 120 | 2.894.715 | 266 | 27.498 |
| EF37 | LR | VREf37-barcode06 | 683.688.059 | 10.576 | 178 | 3.093.572 | 4 | 2.893.407 |
| EF40 | SR | EF40_10m_S61 | 405.445.458 | 145 | 145 | 2.922.643 | 487 | 12.982 |
| EF40 | LR | VREf40-barcode07 | 535.989.354 | 10.400 | 112 | 3.200.545 | 5 | 2.853.862 |
| LC | SR | EF-VREK1-25_SR | 286.883.989 | 146 | 96 | 2.905.859 | 335 | 26.621 |
| LC | LR | EFK1_SQK-NBD114-24_barcode01 | 679.089.360 | 11.604 | 204 | 3.272.320 | 6 | 2.874.387 |
| LC | SR | EF-VRE27-25_SR | 335.744.386 | 146 | 116 | 2.834.879 | 265 | 23.671 |
| LC | LR | EF27_SQK-NBD114-24_barcode19 | 540.616.253 | 10.169 | 173 | 3.157.806 | 9 | 1.911.984 |
| LC | SR | EF-VREK3-25_SR | 279.316.280 | 147 | 91 | 3.002.038 | 287 | 27.479 |
| LC | LR | EFK3_SQK-NBD114-24_barcode03 | 832.778.470 | 11.230 | 262 | 3.143.145 | 5 | 2.927.888 |

**Supplementary Table S3.** WGS metrics of raw reads and assemblies of two sequencing techniques (LR – long-read nanopore sequencing and SR – short-read Illumina sequencing). The table includes 19 outbreak isolates, one long-time exposure isolate (EF12-II) and three local control (LC) isolates. For each assembled genome the table includes the number of input bases and the read N50, average coverage of the assembly, assembly length, contigs and N50 of the final assembly.

| **Incubation** | **Strain** | **BA** | | **CHR** | | **OXO** | | **LIO** | | **BMX** | | **KV** | |
| --- | --- | --- | --- | --- | --- | --- | --- | --- | --- | --- | --- | --- | --- |
| 24h | VREfm *vanA* | + |  | + |  | + |  | + |  | + |  | + |  |
|  | VREfm *vanB* | + |  | + |  | ± |  | ± |  | - |  | - |  |
|  | VREfm_styr_ | + | 7/7 | + | 4/7 | - | 0/7 | - | 0/7 | - | 0/7 | - | 0/7 |
|  |  |  |  |  |  |  |  |  |  |  |  |  |  |
| 48h | VREfm *vanA* | + |  | + |  | + |  | + |  | + |  | + |  |
|  | VREfm *vanB* | + |  | + |  | + |  | + |  | ± |  | ± |  |
|  | VREfm_styr_ | + | 7/7 | + | 7/7 | ± | + 1/7  ± 6/7 | - | ± 1/7  - 6/7 | - | ± 3/7  - 4/7 | - | 0/7 |
|  |  |  |  |  |  |  |  |  |  |  |  |  |  |
| 72h | VREfm *van*A | + |  | + |  | + |  | + |  | + |  | + |  |
|  | VREfm *vanB* | + |  | + |  | + |  | + |  | + |  | ± |  |
|  | VREfm_styr_ | + | 7/7 | + | 7/7 | + | + 6/7  ± 1/7 | - | - 4/7  ± 3/7 | ± | ± 5/7  + 1/7 | - | 0/7 |

**Supplementary Table S4.** Growth of different VREfm strains on blood agar (BA), kanamycin–vancomycin agar (KV), and commercial VRE screening media (CHR, CHROMagar VRE; OXO, Brilliance VRE; LIO, CHROMID VRE; BMX, chromID VRE). Results for VREfm_styr_ are based on seven randomly selected non-duplicate isolates. For each medium, the first column shows the majority growth pattern, whereas the second column provides the exact distribution of growth patterns among the seven VREfm_styr_ isolates. +, confluent growth of colonies; ±, growth of single colonies; –, no growth.
